# A patient with albinism and retinitis pigmentosa, a case report

**DOI:** 10.1016/j.ajoc.2024.102068

**Published:** 2024-05-06

**Authors:** Michalis Georgiou, Shaima Awadh Hashem, Michel Michaelides, Joseph G. Chacko, Sami H. Uwaydat

**Affiliations:** a Jones Eye Institute, University of Arkansas for Medical Sciences, Little Rock, AR, USA; b Moorfields Eye Hospital NHS Foundation Trust, City Road, London, EC1V 2PD, UK; c UCL Institute of Ophthalmology, University College London, 11-43 Bath Street, London, EC1V 9EL, UK

**Keywords:** Albinism, TYR, PDE6A, Retinitis pigmentosa, Genetics, Retinal degeneration, Phenotype

## Abstract

**Purpose:**

To present a case of molecularly confirmed oculocutaneous albinism (OCA) and retinitis pigmentosa (RP).

**Observations:**

A 46-year-old male with a lifelong established diagnosis of OCA and baseline best corrected visual acuity (BCVA) of 20/200, presented for worsening visual acuity over the last few years. BCVA was light perception and hand motion at face for the right and left eye, respectively. Fundus exam showed hypopigmented fundi with visible choroidal vessels and blunted foveal reflexes in both eyes. Optical coherence tomography showed foveal hypoplasia and outer retinal degenerative changes not typical of OCA. Fundus autofluorescence (FAF) imaging showed focal areas of decreased signal at the fovea, similar to areas of atrophy in an age matched patient with *PDE6A*-RP. Genetic testing identified a homozygous disease-causing variant in *TYR* c.1467dup, p. (Ala490Cysfs*20) causing OCA, and a homozygous pathogenic variant c.304C > A, p. (Arg102Ser) in *PDE6A* causing autosomal recessive RP.

**Conclusions and importance:**

This is the first report of a patient with OCA and RP. The lack of pigmentary changes can make the diagnosis of RP challenging in patients with albinism. FAF can show features suggestive of RP and genetic testing can establish the diagnosis. The findings described herein may help physicians diagnose an extremely rare phenotype.

## 1 Introduction

Retinitis pigmentosa (RP) is the most common inherited retinal degeneration phenotype and is characterized by nyctalopia and gradual constriction of the visual field, with eventual loss of central vision, progressing to legal blindness. RP can be inherited as an autosomal dominant (AD), autosomal recessive (AR) or X-linked (XL) trait.^1^ Oculocutaneous albinism (OCA) is a group of genetic disorders characterized by general skin, hair and retinal hypopigmentation, most commonly inherited as an AR condition.^2^ Major and minor criteria for the diagnosis of OCA have been recently proposed. Major criteria include: (1) grade 2 or more foveal hypoplasia, (2) optic nerve misrouting, and (3) ocular hypopigmentation, with either iris translucency or grade 2 or more fundus hypopigmentation. Minor criteria include: (1) nystagmus, (2) hypopigmentation of skin and hair, (3) grade 1 fundus hypopigmentation, and (4) grade 1 foveal hypoplasia. Kruijt et al. proposed that 3 major criteria or 2 major and 2 minor criteria are necessary for the diagnosis OCA. In the presence of a molecular diagnosis, 1 major criterion or 2 minor criteria is considered sufficient.^2^

Herein we report a molecularly confirmed patient with OCA and RP. We describe in detail the presentation of this rare entity.

## 2 Case report

A 46-year-old white male with lifelong established clinical diagnosis of OCA, without molecular confirmation, presented to our ocular genetics clinic (Jones Eye Institute, University of Arkansas Medical Science, Little Rock, AR) for worsening vision. Based on the referral records, the patient had a best corrected visual acuity (BCVA) of 20/200 for both eyes for most of his life, and nystagmus since birth. Patient reported progressive vision loss over the last few years, prompting the referral.

**Fig. 1:**
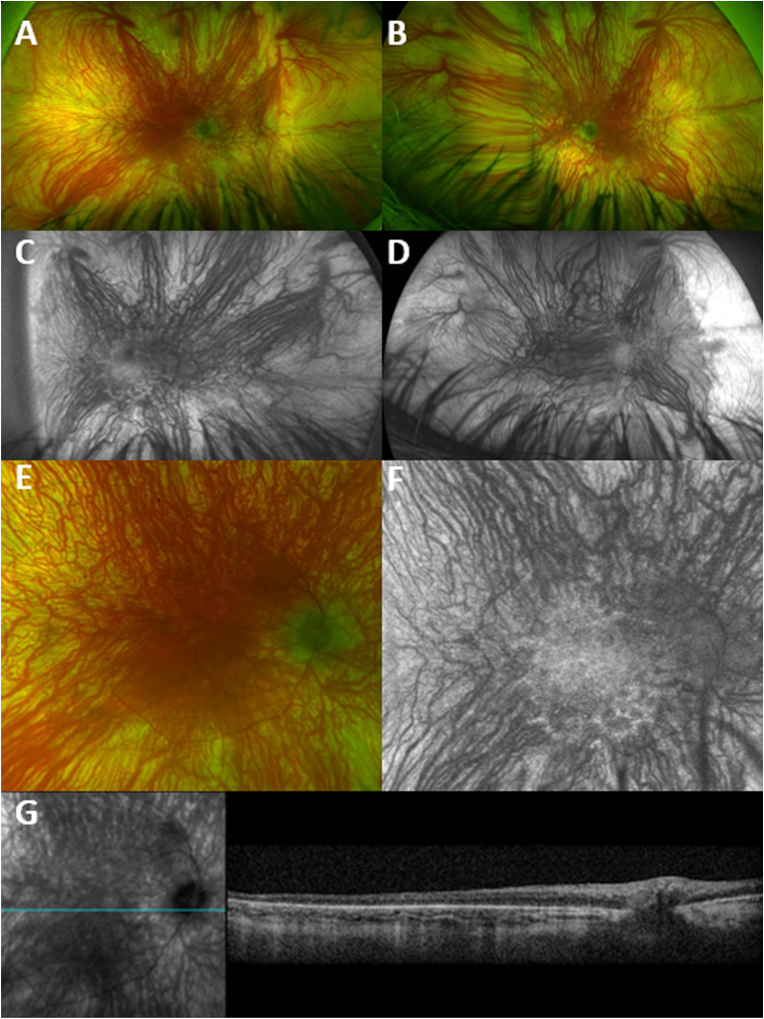
Multimodal Macular Imaging of a Patient with Retinitis Pigmentosa and Albinism. Pseudocolor fundus photographs of the right (A) and left (B) eye of a patient with *PDE6A*-retinitis pigmentosa and *TYR*-albinism, with bilateral blunted reflex, attenuated vessels, small and gray appearing disks. Fundus autofluorescence (FAF) of the right (C) and left (D) eye of the same patient with profound choroidal vessel show. Spotty areas of decreased signal are visible within the arcades, easier to appreciate at the inferior arcade of the right eye (C), due to better imaging quality, which are not usually present in patients with albinism. (E) Pseudocolor fundus magnified image of the macula. (F) Magnified FAF imaging showing profound choroidal vessel show, and patchy areas of signal loss inside the vascular arcades. (G) Infrared image showing the location of the transfoveal optical coherence tomography (OCT) scan. OCT shows grade 4 foveal hypoplasia, with absence of the normal widening of the outer nuclear layer, a finding common in albinism. In contrast to albinism, the ellipsoid zone is disrupted/attenuated. Retinal imaging was challenging due to nystagmus and associated artefacts.

Pupils were equal, 4mm and minimally reactive. BCVA was light perception (LP) and hand motion (HM) at face for the right and left eye respectively, not improving with pinhole or refraction. Patient could not identify any of the Ishihara colour plates due to the severely reduced vision. Intraocular pressure was 15/16 mmHg. Confrontational visual fields could not be assessed. Ocular motility was intact, and severe nystagmus was noted. Examination of the anterior segment was remarkable for 360° iris transilluminations and mild posterior subcapsular cataract in both eyes. Dilated fundoscopy (DFE) revealed a blunted foveal reflex, blonde fundi and visible choroidal vessels, with generalised retinal vessel attenuation (Fig. 1A–B). Optic discs had a gray appearance and were sharp with 0.1 cup/disc ratio in both eyes.

## 3 Retinal imaging

Ultra widefield fundus autofluorescence (FAF, Optos Silverstone, Marlborough, MA, USA) imaging was significant for extensive choroidal show, attenuated retinal vessels and decreased signal within the arcades, which typically are not findings in patients with OCA (Fig. 1C–D). Optical coherence tomography (OCT, Spectralis, Heidelberg Engineering Franklin, MA, USA) showed grade 4 foveal hypoplasia, Fig. 1G. Ellipsoid zone (EZ) was disrupted and attenuated, which is not a typical presentation of OCA. Retinal imaging was challenging due to nystagmus.

## 4 Genetic testing

The patient underwent genetic panel testing, which included ssequence analysis and deletion/duplication testing of 314 genes (Blueprint Genetics, My Retina Tracker Program Panel, version 10, Jul 10, 2023), and was found to be homozygous for disease causing variants (classified as pathogenic) in the *TYR* and *PDE6A* genes. Patient was homozygous for the *TYR* (NM_000372.5): c.1467dup, p. (Ala490Cysfs*20) variant that generates a frameshift resulting in a premature stop codon in the last exon, and for the *PDE6A* (NM_000440.3): c.304C > A, p. (Arg102Ser) missense variant. There are other published reports of the same phenotype caused by other truncating variants 3′ side in the last exon of the *TYR* gene (NM_000372.5 (TYR):c.1501dup (p.Arg501fs)). This can support moderate to strong evidence of the pathogenicity based on the ACMG guidelines.^3^ There are several published reports of the same *PDE6A* gene variant in multiple families, and the variant is classified as pathogenic.

## 5 Controls

Given the inherent variability of presentation in patients with inherited retinal diseases, we performed a search on the genetic database of the Jones Eye Institute, University of Arkansas Medical Science, Little Rock, AR, and of the Moorfields Eye Hospital (MEH), London, UK, for patients with isolated *PDE6A*-RP or *TYR*-albinism with the same variants and of similar age.

**Fig. 2:**
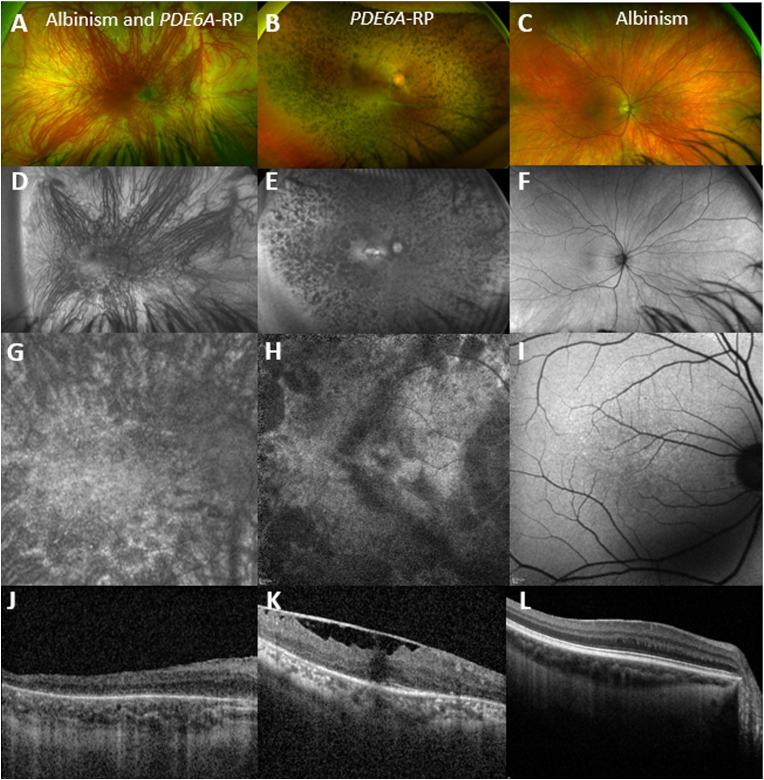
Imaging Comparison with a Control with Retinitis Pigmentosa and a Control with Albinism. On the left column; retinal imaging of a 49-year-old male with *TYR*-albinism and *PDE6A*-retinitis pigmentosa (RP). Middle column depicts retinal imaging of a 47-year-old female with the same homozygous pathogenic *PDE6A*-RP variant and isolated RP. Right column depicts retinal imaging of a 49-year-old male with *TYR*-albinism. (A–C) Widefield pseudocolor fundus images. (D–E) fundus autofluorescence (FAF) images with (G–I) FAF images of the macula at greater magnification, and (J–L) corresponding transfoveal optical coherence tomography (OCT). (G) and (H) show similar pattern of signal changes at the arcades in both patients and vessel attenuation, with the patient with albinism having also visible choroidal vasculature in the periphery. In contrast, patients with isolated albinism do not have the areas of decreased signal on FAF (I), but have foveal hypoplasia on OCT (L). (J) OCT showed foveal hypoplasia and disrupted ellipsoid zone for the patient with albinism and RP. The patient with isolated RP had better preserved foveal ellipsoid zone compared to the patient with albinism and RP, and ERM. RP: retinitis pigmentosa.

We identified a matching 48 year old female control in the MEH database, homozygous for the same *PDE6A* variant. Age of disease onset was 12 years old, presenting with nyctalopia. BCVA was 0.3 LogMAR (20/40) and 0.5 LogMAR (20/60). Patient had normally pigmented iris, pupils were equal and reactive, and trace posterior subcapsular changes were present. DFE showed midperipheral bone-spicule-like retinal pigmentary changes and atrophic changes extending from the periphery to the vascular arcades (Fig. 2 B). Vessel attenuation, epiretinal membrane, and waxy disc pallor were also observed. Widefield FAF showed patchy areas of decreased signal in the mid-periphery and up to the vascular arcades (Fig. 2 E). Macular FAF showed similar pattern of signal changes to the patient with OCA and RP (Fig. 2 G and H). OCT showed better preserved foveal EZ compared to the patient with OCA and RP (Fig. 2 J and K). All findings were similar between the right and the left eye.

We also identified a matching 48 year old male control in the MEH database, compound heterozygous for the same *TYR* variant c.1467dup, p. (Ala490Cysfs*20), and the c.1366+4A > G *TYR* variant. Age of disease onset was 5 years old, presenting with nystagmus. BCVA was 0.3 LogMAR (20/40) and 0.5 LogMAR (20/60). Patient had 360° iris transilluminations. DFE showed foveal hypoplasia, blonde fundi and choroidal show, to a lesser extent than the patient with concurrent RP (Fig. 2C). Widefield FAF showed mostly preserved signal (Fig. 2F), and the macula showed loss of normally decreased signal (Fig. 2 I), a finding also present in our case with OCA and RP (Fig. 2 G). OCT showed better preserved foveal EZ compared to the patient with OCA and RP, and the patient with isolated RP (Fig. 2 L). All findings were similar among the right and the left eye.

## 6 Discussion

OCA is characterized by the reduction or absence of melanin in the skin, hair, and eyes, and consequent photosensitivity, high risk of skin cancer, and reduced visual acuity and nystagmus. The non-syndromic OCA is inherited in an AR manner and is mainly due to disease-causing variants in four genes: *TYR* (OCA1), *OCA2* (OCA2), *TYRP1* (OCA3), and *SLC45A2* (OCA4).^4^ The prevalence of OCA1 is 1:40,000 (ORPHA79431). The *TYR* (MIM *606,933) gene encodes tyrosinase, which participates in the catalysis of the conversion of tyrosine to melanin. OCA caused by variants in the *TYR* gene is divided clinically into two types: type IA is characterized by a complete lack of tyrosinase activity due to the production of an inactive enzyme, and type IB is characterized by reduced activity of tyrosinase. Our patient was homozygous for the *TYR* gene c.1467dup, p. (Ala490Cysfs*20) variant that generates a frameshift resulting in a premature stop codon in the last exon. This is not expected to result in nonsense mediated decay, although it is predicted to truncate the protein, and may result in disrupted protein function. In the literature, this variant has been reported as heterozygous together with other disease causing *TYR* variants, causing OCA.^5^, ^6^, ^7^, ^8^ It is submitted to ClinVar (variation ID 3803) and is listed in the Albinism Database. The patient met almost all the previously suggested clinical criteria for the diagnosis of albinism including; grade 4 foveal hypoplasia, ocular hypopigmentation (both fundus and iris), nystagmus, and hypopigmentation of skin and hair.^2^ No ERG was undertaken and no optic nerve misrouting can be proven, which is a limitation of our study.

The *PDE6A* gene (OMIM *180,071) encodes cGMP-specific phosphodiesterase 6A of the retinal rod cells. This protein is heterotetrametric and consists of alpha, beta (PDE6B, OMIM *180,072), and 2 gamma subunits (PDE6G, OMIM *180,073), and is involved in the processing, transmission and amplification of the visual signal and causes autosomal recessive RP.^1^ *PDE6A* c.304C > A, p. (Arg102Ser) has previously been reported both in compound heterozygous and homozygous.^9^, ^10^, ^11^, ^12^ The codon appears to be highly conserved. We previously reported a missense variant affecting the same codon in the *PDE6C* gene causing achromatopsia.^13^ *PDE6B-*RP is the target of gene therapy trial (NCT03328130),^14^ with the results of the trial being relevant to future therapeutic prospects for *PDE6A*-RP.

Our patient did not exhibit the classic phenotypic features of RP,^15^ due to the concurrent presentation of OCA. The worsening of his vision was not expected in the clinical course of OCA. High degree of clinical suspicion is needed for patients with rare diseases for possible concurrent pathology. The FAF and OCT imaging were the most informative for the concurrent *PDE6A*-RP, even though the changes were subtle and the imaging quality was poor due to nystagmus. The vision of our patient was worse than age matched patients with either isolated *PDE6A-*RP or isolated *TYR*-OCA, likely due to the dual pathology. We have previously reported similar findings of worse outcomes with dual pathology for patients with albinism and proliferative diabetic retinopathy.^16^ Similarities in the FAF phenotypes can be tracked among the three phenotypes of the current report (Fig. 2). The patient with OCA and RP had areas of decreased signal similar to the patient with RP along the arcades, and loss of the normally decreased foveal signal similar to the patient with isolated OCA. Our patient is unlikely to benefit from any gene specific treatment in the future due to his dual pathology. Vision rehabilitation and cataract surgery may help maximize his potential acuity.^17^

## 7 Conclusions

We present a well-documented molecularly confirmed case of a patient with *TYR*-OCA and *PDE6A*-RP. The case highlights the need of clinical awareness for rare diseases and the usefulness of genetic testing.

## 8 Patient consent

Written consent to publish those cases has not been obtained. This report does not contain any personal identifying information.

## Funding

None.

## 9 Authorship

All authors attest that they meet the current ICMJE criteria for authorship.

## CRediT authorship contribution statement

**Michalis Georgiou:** Methodology, Investigation, Funding acquisition, Formal analysis, Data curation, Conceptualization. **Shaima Awadh Hashem:** Formal analysis, Data curation. **Michel Michaelides:** Writing – review & editing, Investigation, Data curation. **Joseph G. Chacko:** Writing – review & editing, Writing – original draft, Supervision, Data curation, Conceptualization. **Sami H. Uwaydat:** Writing – review & editing, Writing – original draft, Supervision, Investigation.

## Declaration of competing interest

The authors declare that they have no known competing financial interests or personal relationships that could have appeared to influence the work reported in this paper.
